# In Vitro Activity of Imipenem/Relebactam and Ceftolozane/Tazobactam Against Multidrug-Resistant *Klebsiella pneumoniae*: A Retrospective Single-Center Study in China

**DOI:** 10.3390/antibiotics15100947

**Published:** 2026-09-23

**Authors:** Juan Yi, Chao Liu, Ping Yang, Bowen Wang, Jiajia Zheng, Ning Shen

**Affiliations:** 1 Institute of Medical Technology, Peking University Health Science Center, Beijing 100191, China; yijuan0329@163.com; 2 Department of Pulmonary and Critical Care Medicine, Peking University Third Hospital, Beijing 100191, China; ypgoafee@163.com; 3 Department of Infectious Disease, Peking University Third Hospital, Beijing 100191, China; liuchao3619@163.com (C.L.); wbw18103929808@163.com (B.W.); 4 Department of Laboratory Medicine, Peking University Third Hospital, Beijing 100191, China; zhengjiajia@bjmu.edu.cn; 5 Department of Respiratory and Critical Care Medicine, Peking University Third Hospital, Beijing 100191, China

**Keywords:** multidrug-resistant *Klebsiella pneumoniae*, hypervirulent *Klebsiella pneumoniae*, imipenem-relebactam, ceftolozane-tazobactam

## Abstract

Background: Multidrug-resistant *Klebsiella pneumoniae* (MDR-KP) is a global health threat with limited treatment options. Comparative in vitro data for imipenem-relebactam (I/R) and ceftolozane-tazobactam (C/T) across diverse MDR-KP clinical populations remain limited. Methods: Susceptibility to I/R and C/T was determined by Etest. Whole-genome sequencing was used to identify sequence types, resistance genes, and virulence genes, with hypervirulent *K. pneumoniae* (hvKP) defined by genomic criteria. Results: A total of 312 nonduplicate MDR-KP clinical isolates, including 70.8% carbapenem-resistant *K. pneumoniae* (CR-KP), 56.7% difficult-to-treat resistant *K. pneumoniae* (DTR-KP), and 28.2% carbapenem-resistant hvKP (CR-hvKP), were retrospectively analyzed. I/R resistance was low across all subgroups (overall 3.8%, CR-KP 5.0%, DTR-KP 6.2%, CR-hvKP 2.3%) and across specimen types (0–9.6%; blood 3.8%). In contrast, C/T resistance was higher (overall 57.7%, CR-KP 79.2%, DTR-KP 91.0%, CR-hvKP 84.1%), with the highest rate in respiratory isolates (63.0%), followed by blood (57.7%) and urine (50.0%). Among extended-spectrum β-lactamase (ESBL)-producing isolates, I/R and C/T resistance rates were 2.1% and 12.4%, respectively. Although C/T resistance was higher in hvKP than in classic *K. pneumoniae* in unadjusted analysis, hvKP was not independently associated with increased C/T resistance after adjustment. Instead, ST11, ST15, ST307, *bla*_KPC-2_, and *bla*_NDM-1_ were independently associated with C/T resistance. For I/R, *bla*_NDM-1_ was associated with resistance. Conclusions: I/R was active across MDR-KP subgroups including CR-hvKP and DTR-KP. C/T worked mainly against ESBL-producers. These findings highlight the importance of resistance genotyping when interpreting susceptibility to these agents.

## 1. Introduction

*Klebsiella pneumoniae* poses a significant threat to global public health [1]. The dissemination of multidrug-resistant *K. pneumoniae* (MDR-KP) is associated with limited therapeutic options and high mortality rates [2,3]. Notably, the convergence of hypervirulence and multidrug resistance further complicates the management of *K. pneumoniae* infections [4,5]. In China, this convergence is especially prominent among high-risk sequence type 11 (ST11) clones [6].

Therapeutic options for MDR-KP infection remain limited [7,8]. Several β-lactam/β-lactamase inhibitor (BL/BLI) combinations, including imipenem-relebactam (I/R) and ceftolozane-tazobactam (C/T), have been used for the treatment of MDR Gram-negative infections [9,10]. C/T retains activity against extended-spectrum β-lactamase (ESBL)-producing *Enterobacterales* [11], whereas I/R has demonstrated activity against carbapenem-resistant *Enterobacterales* (CRE), particularly *Klebsiella pneumoniae* carbapenemase (KPC)-producing isolates [12]. Large-scale surveillance studies have shown I/R to be highly active against KPC-producing *K. pneumoniae* (KPC-KP). Guo et al. [13] reported an I/R resistance rate of only 0.8% among 782 KPC-KP isolates across China. Palomba et al. [14] found 98% I/R susceptibility in a 603-strain KPC-KP cohort from Italy. US surveillance data from 2020–2024 demonstrated high I/R activity against KPC-producing isolates, but substantially lower activity against New Delhi metallo-β-lactamase (NDM)-producing isolates [12]. However, these studies focused predominantly on KPC-KP and did not systematically examine resistance patterns across the broader MDR-KP population, particularly by specimen source and virulence background.

Three studies from Sichuan Province examined carbapenem-resistant hypervirulent *K. pneumoniae* (CR-hvKP) isolates but reported inconsistent results. C/T resistance was uniformly high, ranging from 81.1% to 99.1% [15], while I/R susceptibility ranged from 57.5% to over 90% [16,17], with the lower estimate contrasting with the >98% susceptibility reported in large multicenter cohorts. These studies were limited by small sample sizes and lacked data on multidrug-resistant hypervirulent *K. pneumoniae* (MDR-hvKP). Although real-world data support I/R for lower respiratory tract infections [9], its in vitro activity across different specimen sources has not been systematically compared.

In this study, we evaluated the in vitro activity of I/R and C/T against MDR-KP clinical isolates and investigated their molecular epidemiological characteristics, with stratified analyses by specimen source and virulence background. I/R showed potent activity across all MDR-KP subgroups, including carbapenem-resistant *K. pneumoniae* (CR-KP), difficult-to-treat resistant *K. pneumoniae* (DTR-KP), and CR-hvKP, with an overall resistance rate of only 3.8%. In contrast, C/T had limited activity against these subgroups, with resistance rates exceeding 57% and notable variations across specimen types.

## 2. Results

### 2.1. Characteristics of the MDR-KP Isolates

A total of 312 nonduplicate clinical isolates of *K. pneumoniae* were included in this study. The collection comprised isolates from various specimen types: respiratory samples (59.0%, 184/312), urine (16.7%, 52/312), blood (8.3%, 26/312), and others (16.0%, 50/312) (Figure 1A). With respect to virulence and resistance phenotypes, the collection included 213 classic *K. pneumoniae* (cKP, 68.3%) and 99 genomically defined hypervirulent *K. pneumoniae* (hvKP, 31.7%) isolates (Figure 1B). All 312 isolates were confirmed to be MDR-KP through antimicrobial susceptibility testing. A total of 221 isolates (70.8%) were identified as CR-KP, 177 (56.7%) met the DTR-KP criteria, 97 (31.1%) were classified as ESBL-KP, and 88 (28.2%) were characterized as CR-hvKP (Figure 1B).

### 2.2. Sequence Types and Resistance and Virulence Genes of MDR-KP

Among the 312 MDR-KP isolates, 60 distinct sequence types (STs) were identified (Figure S1). ST11 was the predominant clone, accounting for 152 isolates (48.7%), followed by ST307 (17.6%, 55/312), ST15 (6.4%, 20/312), and ST37 (2.9%, 9/312). Carbapenemase genes were detected in 172 isolates (55.1%), with *bla*_KPC-2_ being the most prevalent (52.6%, 164/312) and predominantly concentrated within the ST11 and ST15 lineages. In contrast, *bla*_NDM-1_ (3.5%, 11/312) was identified across more diverse backgrounds, including ST307 and other STs. ESBL genes were also widely distributed: *bla*_CTX-M-65_ (31.7%, 99/312) was predominantly carried by ST11, whereas *bla*_CTX-M-15_ (16.0%, 50/312) and *bla*_CTX-M-55_ (8.3%, 26/312) were more common in ST307 and ST15 (Figure S1). Other frequent β-lactamase genes included *bla*_SHV-12_ (14.1%, 44/312), *bla*_CTX-M-3_ (6.4%, 20/312), and *bla*_CMY-42_ (6.1%, 19/312).

A total of 99 isolates (31.7%) harbored at least one of the five key virulence markers. Specifically, *iucA* was detected in 98 isolates, *rmpA2* in 80, *peg-344* in 33, *rmpA* in 29, and *iroB* in 11. These virulence determinants were highly enriched in the ST11 cluster (Figure S1). Consequently, 88 isolates (28.2%) carried both carbapenem resistance and hypervirulence genes (CR-hvKP), the vast majority of which belonged to ST11 (93.2%, 82/88).

### 2.3. In Vitro Activity of Antimicrobial Agents Against MDR-KP

The antimicrobial susceptibility testing (AST) results are summarized in Table 1. The resistance rate for I/R was 3.8% (12/312), which was lower than that for carbapenems (57.4–67.9%). In contrast, C/T showed more limited efficacy, with a susceptibility rate of 32.7% (102/312) and a resistance rate exceeding 57% (180/312). In contrast, traditional BL/BLI combinations such as piperacillin/tazobactam (TZP) and cefoperazone/sulbactam (CSL) were largely compromised, with resistance rates exceeding 84.9% (265/312) and 73.4% (229/312), respectively. With respect to C/T, the MIC_50_ and MIC_90_ values were 24 mg/L and ≥256 mg/L, respectively, while I/R was more potent, with values of 0.38 mg/L and 1.5 mg/L, respectively (Figure 2A,B). Among the non-β-lactam agents, amikacin (AMK) displayed moderate activity, with a susceptibility rate of 52.9% (165/312), whereas levofloxacin (LVX) and minocycline (MNO) showed poor efficacy, with susceptibility rates of only 1.9% (6/312) and 9.6% (30/312), respectively.

I/R exhibited consistently low resistance across specimen types (Figure 3A), with rates of 9.6% (5/52) in urine, 3.8% (1/26) in blood, 3.3% (6/184) in respiratory samples, and 0% in other sources. Conversely, C/T demonstrated high resistance frequencies, peaking in respiratory isolates (63.0%, 116/184), followed by blood (57.7%, 15/26), urine (50.0%, 26/52), and other sources 46.0% (23/50). Overall, both agents performed better than traditional carbapenems and cephalosporins, whose resistance rates often exceeded 60% to 90% in respiratory and blood samples.

As shown in Figure 3B, antimicrobial resistance rates varied across different subgroups. Overall, high resistance rates were observed for FEP, CAZ, TZP, IPM, MEM, ETP, and LVX, particularly among CR-KP and DTR-KP isolates, where resistance frequently approached or reached 100%. Notably, AMK, MNO, and SXT showed variable resistance patterns, with rates exceeding 50% in most subgroups. Importantly, CZA remained highly active across all subgroups, with resistance rates consistently below 30%. I/R demonstrated sustained in vitro activity against both CR-KP (5.0%, 11/221) and DTR-KP (6.2%, 11/177) isolates, whereas C/T exhibited high resistance levels, reaching 79.2% (175/221) in the CR-KP group and 91.0% (161/177) in the DTR-KP group. Specifically, I/R yielded MIC_50/90_ values of 0.5/1.5 mg/L in the CR-KP group (Figure 2D) and 0.75/2 mg/L in the DTR-KP group (Figure 2F). In contrast, the C/T MIC_50/90_ values were greater, reaching 48/≥256 mg/L and 64/≥256 mg/L, respectively (Figure 2C,E).

In the ESBL-KP subgroup, C/T exhibited potent activity with a resistance rate of 12.4% (12/97) (Figure 3B). It was lower than those of traditional agents, such as CSL (40.2%, 39/97) and TZP (64.9%, 63/97), as well as ETP (23.7%, 23/97), but remained higher than those of MEM (6.2%, 6/97), IPM (2.1%, 2/97), and CZA (4.1%, 4/97). I/R remained highly potent in this subgroup, with a resistance rate of only 2.1% (2/97). MIC distributions revealed that C/T yielded MIC_50_ and MIC_90_ values of 1.5 mg/L and 24 mg/L, respectively, whereas I/R displayed higher potency, with values of 0.125 mg/L and 0.25 mg/L, respectively (Figure 2G,H).

### 2.4. The Activity of C/T and I/R Against hvKP

The in vitro activity of C/T and I/R is shown in Figure 4. Among MDR-KP isolates, C/T resistance was significantly more frequent in hvKP than in cKP isolates (76.8% vs. 48.8%, *p* < 0.001), but this difference was not consistent across other subgroups. In DTR-KP isolates, by contrast, cKP isolates showed higher resistance than hvKP isolates to both C/T (95.7% vs. 85.9%, *p* = 0.024) and I/R (9.8% vs. 2.4%, *p* = 0.041) (Table S1). Among ESBL-KP and CR-KP isolates, however, C/T resistance rates were comparable between cKP and hvKP isolates. I/R resistance was comparable between cKP and hvKP isolates among ESBL-KP, CR-KP and MDR-KP isolates. I/R showed substantially lower resistance rates than C/T across the hvKP subgroups. I/R resistance was observed in 2.0% (2/99) of MDR-hvKP, 2.3% (2/88) of CR-hvKP, and 2.4% (2/85) of DTR-hvKP isolates. In contrast, C/T resistance occurred in 76.8% (76/99) of MDR-hvKP, 84.1% (74/88) of CR-hvKP, 85.9% (73/85) of DTR-hvKP, and 14.3% (1/7) of ESBL-hvKP isolates (Figure S2).

Given the strong clonal structure of the collection, multivariable logistic regression analyses were performed to assess whether the observed differences according to hvKP phenotype persisted after adjustment for ST lineage, *bla*_KPC-2_ and *bla*_NDM-1_ carriage, ESBL genes, and specimen source (Table 2). After adjustment, hvKP was not associated with increased C/T resistance (aOR, 0.37 [0.10–1.20]; *p* = 0.117). In contrast, ST11 (aOR, 16.18 [3.56–77.11]; *p* < 0.001), ST15 (aOR, 19.43 [2.62–189.83]; *p* = 0.005), ST307 (aOR, 3.87 [1.23–14.03]; *p* = 0.026), *bla*_KPC-2_ carriage (aOR, 25.98 [7.07–113.35]; *p* < 0.001), and *bla*_NDM-1_ carriage (aOR, 115.46 [15.70–2529.49]; *p* < 0.001) were independently associated with C/T resistance. ESBL genes (aOR, 0.99 [0.40–2.45]; *p* = 0.990) and specimen source (aOR, 1.71 [0.73–4.10]; *p* = 0.217) were not independently associated with C/T resistance. Owing to the small number of *bla*_NDM-1_-carrying isolates and the correspondingly wide confidence interval, the adjusted estimate for *bla*_NDM-1_ should be interpreted cautiously.

For I/R resistance, hvKP was not independently associated with resistance before or after adjustment. *bla*_NDM-1_ carriage (aOR, 56.62 [11.46–383.20]; *p* < 0.001) was associated with I/R resistance, whereas ST11, ST15, ST307, *bla*_KPC-2_, ESBL genes, and specimen source were not independently associated with I/R resistance. The direction of the association for ST11 reversed between univariable (OR 0.25, 95% CI 0.06–0.85; *p* = 0.026) and multivariable analysis, most likely owing to collinearity with *bla*_KPC-2_ (Table S2).

### 2.5. Antimicrobial Resistance Profiles of I/R- and C/T-Resistant Isolates

The resistance profiles varied by STs and β-lactamase background as demonstrated in Figure 3C and Table 3. High-level C/T resistance was predominant in ST11 (89.5%, 136/152) and ST15 (90.0%, 18/20) but lower in ST307 (23.6%, 13/55). I/R maintained high activity across major endemic clones, with resistance rates not exceeding 5.0% for the STs shown. ST11 isolates exhibited nearly universal resistance to carbapenems (>92%), whereas ST307 showed lower resistance to MEM (14.5%, 8/55) and IPM (5.5%, 3/55). CZA resistance remained low in both lineages, but was slightly higher in ST11 (27.6%, 42/152) than in ST307 (5.5%, 3/55).

Across carbapenemase-producing isolates, I/R resistance was 5.2% (9/172), whereas nearly all the isolates were resistant to C/T (93.6%, 161/172). I/R retained high activity against *bla*_KPC-2_-carrying isolates, with a susceptibility rate of 87.2% (143/164). In contrast, reduced activity was observed in *bla*_NDM-1_-positive isolates, with an I/R resistance of 54.5% (6/11), whereas the C/T resistance rate reached 100.0% for most *bla*_NDM-1_-carrying combinations. The most common genotype was coharboring *bla*_CTX-M-65_ and *bla*_KPC-2_, with I/R and C/T resistance rates of 4.0% (2/50) and 90.0% (45/50), respectively. Isolates carrying *bla*_KPC-2_ alone were fully susceptible to I/R but exhibited high resistance to C/T (90.3%, 28/31). Similarly, combinations of *bla*_KPC-2_ with *bla*_CTX-M-15_, *bla*_SHV-12_, or *bla*_CMY-42_ plus *bla*_CTX-M-65_ conferred high C/T resistance (93.3–100.0%) while maintaining full susceptibility to I/R. In isolates without detectable ESBL or carbapenemase genes, resistance rates were 2.3% (1/43) for I/R and 16.3% (7/43) for C/T.

Nine of the 12 I/R-resistant isolates, carried at least one carbapenemase gene. The remaining three isolates, belonging to ST15, ST20 and ST37, carried no carbapenemase gene. Porin defects were detected in six isolates. All six carried a truncated OmpK35 that retained only 7 to 21 percent of the full-length amino acid sequence from the start codon, and all six concurrently carried an altered OmpK36, which was the Gly-Asp insertion in extracellular loop 3 in five isolates and a truncation to 9 percent of the amino acid sequence in the remaining isolate. The characteristics of all 12 isolates are summarised in Table S3.

## 3. Discussion

The dissemination of MDR-KP, particularly strains co-exhibiting carbapenem resistance and hypervirulence genes, which threatens global antimicrobial therapy [18,19]. In this single-center retrospective study, we evaluated the in vitro activity of C/T and I/R against MDR-KP clinical isolates, with particular emphasis on specimen source and genomically defined hypervirulence. I/R demonstrated consistently low resistance across all MDR-KP subgroups and specimen types, whereas C/T resistance was higher and varied by specimen source. These data complement recent multicenter reports from China [13] and Italy [14], which focused mainly on KPC-KP without systematic stratification by specimen source or virulence background. Notably, the I/R resistance rate among CR-hvKP isolates in our cohort (2.3%) was lower than the 6.6% reported in a recent Sichuan study [16], suggesting regional variation in I/R activity against CR-hvKP.

Despite the high prevalence of CR-KP (70.8%) and DTR-KP (56.7%) in this MDR-KP collection, I/R resistance was uncommon (3.8%, 12/312), and I/R showed high in vitro activity (89.7% susceptibility, 280/312). Notably, I/R retained high in vitro activity against CR-KP isolates (resistance 5.0%), compared with 24.4% for CZA. The high susceptibility was consistent with the predominance of *bla*_KPC-2_, which accounted for more than half of the isolates and represented the major carbapenem mechanism in this cohort. Relebactam restores imipenem activity against Ambler class A and class C β-lactamases, including *bla*_KPC-2_ [20]. In contrast, isolates harboring *bla*_NDM-1_ were largely resistant to I/R, consistent with the inability of relebactam to inhibit metallo-β-lactamases [12,20,21]. Among the 12 I/R-resistant isolates, *bla*_NDM-1_ was detected in six isolates, whereas *bla*_KPC-2_ was present in four isolates. Complementing these observations, a recent large-scale study of clinical KPC-KP isolates in China further confirmed the superior in vitro efficacy of I/R over CZA and identifying distinct resistance mechanisms involving *bla*_KPC_ mutations, ompK36 alterations, and elevated *bla*_KPC_ expression [13]. OmpK35/OmpK36 alterations were also observed in several resistant isolates. Neither efflux pump activity nor quantitative β-lactamase expression was assessed in this study; therefore, the mechanism of I/R resistance in the three isolates that carried no carbapenemase gene and showed no OmpK alteration remains unexplained and requires further investigation. Within the DTR-KP subgroup, I/R resistance was significantly more frequent in cKP than in hvKP isolates, whereas no significant differences were observed in the other subgroups or comparisons. In univariable and multivariable Firth logistic regression, only *bla*_NDM-1_ was independently associated with I/R resistance (aOR 56.62, *p* < 0.001). However, only 12 I/R-resistant isolates were available; despite the use of Firth penalized regression (EPV 1.5), the adjusted estimates for I/R resistance remain exploratory. These findings highlight the importance of carbapenemase genotype, particularly *bla*_NDM-1_ carriage, in interpreting I/R susceptibility.

The emergence of CR-hvKP poses a serious clinical threat [22]. In this study, more than one-quarter of the isolates were defined as CR-hvKP, predominantly belonging to the ST11 lineage. Consistent with previous reports [15], the CR-hvKP isolates were highly resistant to carbapenems, FEP, CAZ, LVX, TZP, and CSL (Figure S2). In contrast, resistance to I/R remained rare among the CR-hvKP isolates (2.3%, 2/88). I/R therefore showed strong in vitro activity against CR-hvKP isolates and may represent a candidate option pending clinical evaluation and susceptibility confirmation.

C/T demonstrated good in vitro activity against ESBL-KP isolates, but limited activity against CR-KP. Resistance rates were 57.7% (180/312) among MDR-KP isolates and 79.2% (175/221) among CR-KP isolates, whereas the ESBL-KP subgroup had a resistance rate of 12.4% (12/97), consistent with previous reports for third-generation cephalosporin-nonsusceptible *K. pneumoniae* (71.8–88.7% susceptibility) [23]. Previous surveillance studies reported susceptibility rates of 49.1% in Europe [24], 56.6% in Latin America [25], and 69.4% in the Asia-Pacific region [26]. In univariable analysis, C/T resistance was more common among hvKP isolates than among cKPs (OR 3.46, 95% CI 2.05–6.03, *p* < 0.001), and resistance reached 84.1% (74/88) in CR-hvKP, consistent with the 81.1–99.1% C/T resistance reported in CR-hvKP from Sichuan [15]. However, after adjustment for sequence type, carbapenemase genes, ESBL genes, and specimen source, hvKP was no longer independently associated with C/T resistance (aOR 0.37, *p* = 0.117). Instead, ST11 (aOR 16.18, *p* < 0.001), ST15 (aOR 19.43, *p* = 0.005), ST307 (aOR 3.87, *p* = 0.026), *bla*_KPC-2_ (aOR 25.98, *p* < 0.001), and *bla*_NDM-1_ (aOR 115.46, *p* < 0.001) remained independently associated with C/T resistance. These findings were consistent with the inhibitory spectrum of C/T, which has little inhibitory activity against KPC and NDM carbapenemases [27]. These findings suggest that the apparent association between hvKP and C/T resistance was associated with clonal and resistance-gene background, particularly the predominance of ST11 KPC-KP in China. Therefore, C/T should be used with caution in regions where ST11 KPC-KP clones are prevalent, and molecular resistance profiling may help guide its potential use.

Consistent with earlier findings showing high C/T activity against *bla*_CTX-M-15-like_ (91.4%) and *bla*_CTX-M-14-like_ (97.5%) but lower activity against *bla*_SHV_ (66.7%) [28], this study revealed that the in vitro activity of C/T differed among ESBL genotypes. Among the ESBL-KP isolates, resistance was lowest in isolates carrying *bla*_CTX-M-14_ (0%, 0/9), followed by those carrying *bla*_CTX-M-3_ (5.3%, 1/19) and *bla*_CTX-M-15_ (10.3%, 3/29), whereas resistance was highest among isolates carrying *bla*_CTX-M-55_ (19.2%, 5/26) and *bla*_CTX-M-27_ (20.0%, 1/5). In addition, 16.3% (7/43) of isolates lacking detectable ESBL or carbapenemase genes were resistant to C/T. These results confirm that the activity of C/T is genotype-dependent and support molecular resistance profiling to guide its potential use.

Several limitations of this study should be acknowledged. First, this was a retrospective, single-center study conducted in China, and the collection of MDR-KP isolates was dominated by ST11 and *bla*_KPC-2_-producing strains. Therefore, the findings may be most applicable to settings with similar clonal and resistance-gene distributions and should not be broadly generalized. Second, no patient-level clinical data were collected in this study. Thus, these findings are limited to in vitro susceptibility and genomic characteristics and do not establish clinical efficacy or treatment outcomes. Third, hypervirulence was defined by genomic markers rather than phenotypic or in vivo validation, which may limit virulence characterization. Fourth, I/R and C/T susceptibility was determined by Etest, and gradient diffusion methods may show minor discrepancies compared with reference methods.

## 4. Materials and Methods

### 4.1. Bacterial Isolation and Definitions

MDR-KP isolates were retrospectively collected from Peking University Third Hospital in Beijing, China, between November 2020 and June 2023. Only the first isolate obtained from each patient was included. All the isolates were identified using the VITEK 2 Compact system (bioMérieux, Marcy-l’Étoile, France) and stored at −80 °C. MDR-KP was defined as nonsusceptibility to at least one agent in three or more antimicrobial categories [29]. CR-KP was defined as an isolate that was not susceptible to at least one carbapenem (imipenem, meropenem, or ertapenem). Isolates exhibiting nonsusceptibility to all first-line agents, including fluoroquinolones, carbapenems, and cephalosporins, were classified as DTR-KP [7]. Isolates carrying ESBL genes without carbapenemase genes were classified as ESBL producers [30]. Hypervirulent *K. pneumoniae* (hvKP) was defined as the presence of at least one of the following virulence genes: *rmpA*, *rmpA2*, *peg-344*, *iroB*, or *iucA* [31]. Classic *K. pneumoniae* (cKP) was defined as the absence of these specific virulence genes. Notably, isolates concurrently exhibiting carbapenem nonsusceptibility and hypervirulence genes were defined as CR-hvKP.

### 4.2. Antimicrobial Susceptibility Testing

The susceptibility of all the isolates to I/R and C/T was assessed using Etest (Liofilchem, Roseto degli Abruzzi, Italy). *Escherichia coli* ATCC 25922 and *K. pneumoniae* ATCC 700603 were utilized for quality control. Susceptibility profiles for other antimicrobial agents, including IPM, MEM, ETP, MNO, AMK, FEP, CAZ, LVX, TZP, CSL, CZA, and SXT, were retrieved from the hospital’s laboratory information system (LIS). Strains with complete susceptibility data for IPM, MEM, ETP, MNO, AMK, FEP, CAZ, LVX, TZP, CSL, and SXT were included in the study. These were originally performed using the VITEK 2 Compact system (bioMérieux, France) or disk diffusion (for ETP and CZA). For isolates lacking retrospective CZA results in the LIS, disk diffusion testing (Liofilchem, Roseto degli Abruzzi, Italy) was subsequently performed. AST results were interpreted according to CLSI M100, 34th edition (2024). For I/R, MIC breakpoints were susceptible ≤1/4 mg/L, intermediate 2/4 mg/L, and resistant ≥4/4 mg/L, with relebactam tested at a fixed concentration of 4 mg/L. For C/T, the corresponding breakpoints were susceptible ≤2/4 mg/L, intermediate 4/4 mg/L, and resistant ≥8/4 mg/L. CSL susceptibility was interpreted on the basis of breakpoints proposed in previous studies [32].

### 4.3. Whole-Genome Sequencing and Bioinformatic Analysis

Genomic DNA was extracted from overnight cultures of all the isolates. Paired-end sequencing (2 × 150 bp) was performed on a NovaSeq platform (Illumina, San Diego, CA, USA), with a target depth of at least 100× [33]. The raw data were processed via Fastp for quality control and assembled using SPAdes v3.13 [34]. Resistance genes and sequence types (STs) were identified using Kleborate software v2.3.2 [35]. Virulence genes were annotated by comparison with Virulence Factor Database using BLAST software v.2.2.18.

### 4.4. Statistical Analysis

Statistical analyses were performed using R (version 4.5.3). Categorical variables were compared using the chi-square or Fisher’s exact test, as appropriate. All tests were two-sided, with *p* < 0.05 considered statistically significant. Data visualization was performed using the ggplot2 (version 4.0.3) package.

Univariable logistic regression was first performed for each variable. Multivariable logistic regression was then used to identify factors independently associated with C/T resistance. Covariates included hvKP status (with cKP as the reference category), ESBL genes, *bla*_KPC-2_ and *bla*_NDM-1_ carriage, sequence type (ST11, ST15, ST307, and other), and specimen source (respiratory vs. non-respiratory). With 180 events and 8 degrees of freedom, the events-per-variable (EPV) ratio was 22.5; therefore, standard non-penalized logistic regression was appropriate. Adjusted odds ratios (aORs) and 95% confidence intervals (CIs) were calculated using profile likelihood. For I/R resistance (n = 12 events), Firth’s penalized-likelihood logistic regression was used with the same set of covariates, because standard non-penalized regression would produce unstable estimates under such sparse events. Given the very low number of I/R-resistant isolates, the corresponding adjusted estimates are exploratory and should be interpreted cautiously.

## 5. Conclusions

In conclusion, I/R demonstrates potent in vitro activity against MDR-KP, including hvKP and high-risk clones. The activity of C/T appears to be largely confined to ESBL-producing isolates. These findings highlight the importance of genomic surveillance and susceptibility testing when interpreting the potential activity of novel BL/BLI combinations.

## Figures and Tables

**Figure 1:**
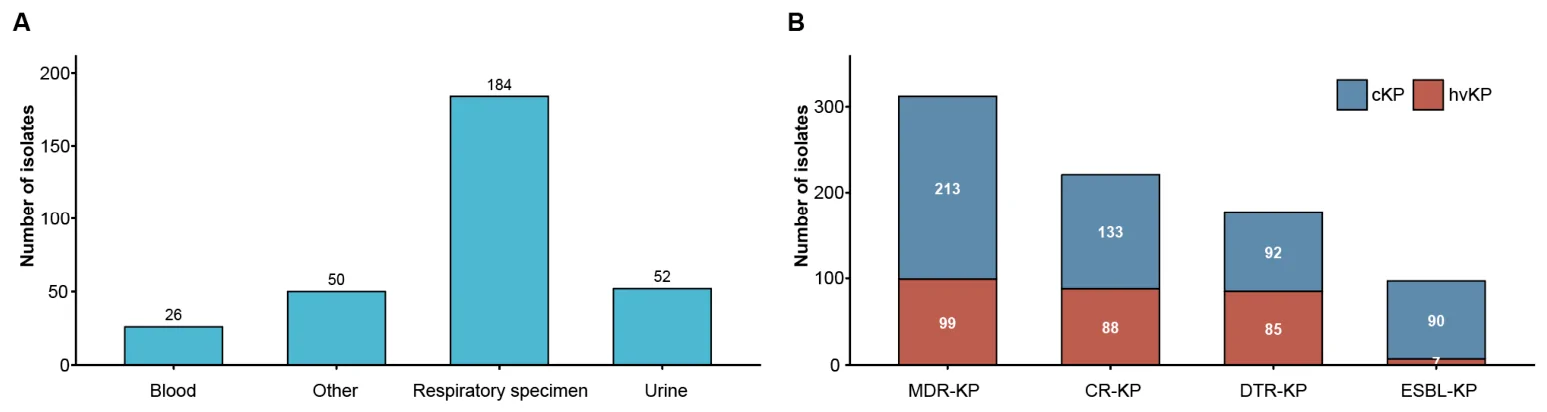
Baseline Characteristics of MDR-KP isolates. (**A**) Distribution of specimen sources. (**B**) Composition of resistance phenotypes and virulence types.

**Figure 2:**
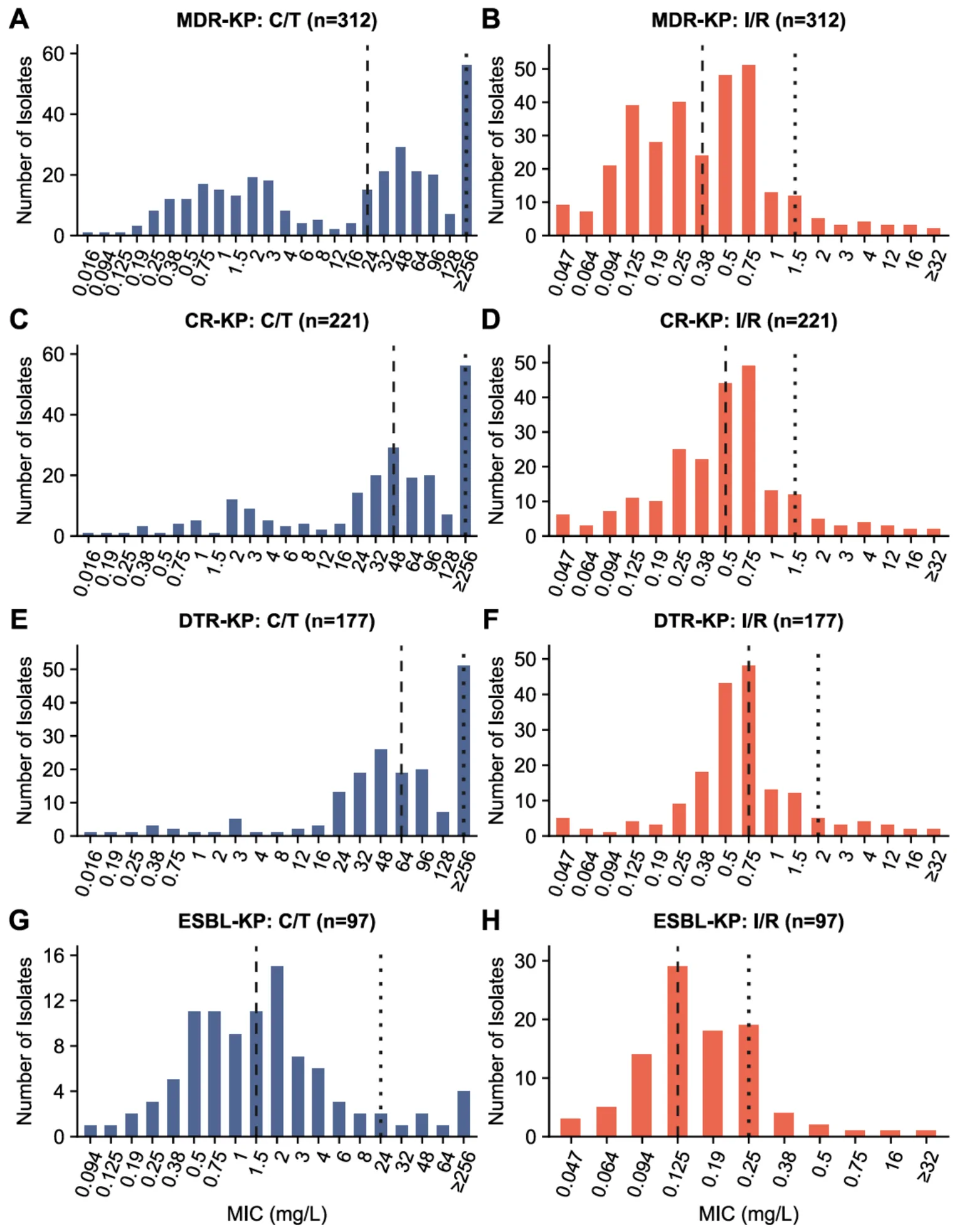
MIC Distributions of C/T and I/R. MIC distributions are shown for MDR-KP (**A**,**B**), CR-KP (**C**,**D**), DTR-KP (**E**,**F**) and ESBL-KP (**G**,**H**) isolates; panels (**A**,**C**,**E**,**G**) show C/T and panels (**B**,**D**,**F**,**H**) show I/R. Vertical dashed and dotted lines indicate the MIC_50_ and MIC_90_ values, respectively. MIC, Minimum Inhibitory Concentration; MDR, multidrug-resistant; CR, carbapenem-resistant; DTR, difficult-to-treat resistance; ESBL, extended-spectrum β-lactamase.

**Figure 3:**
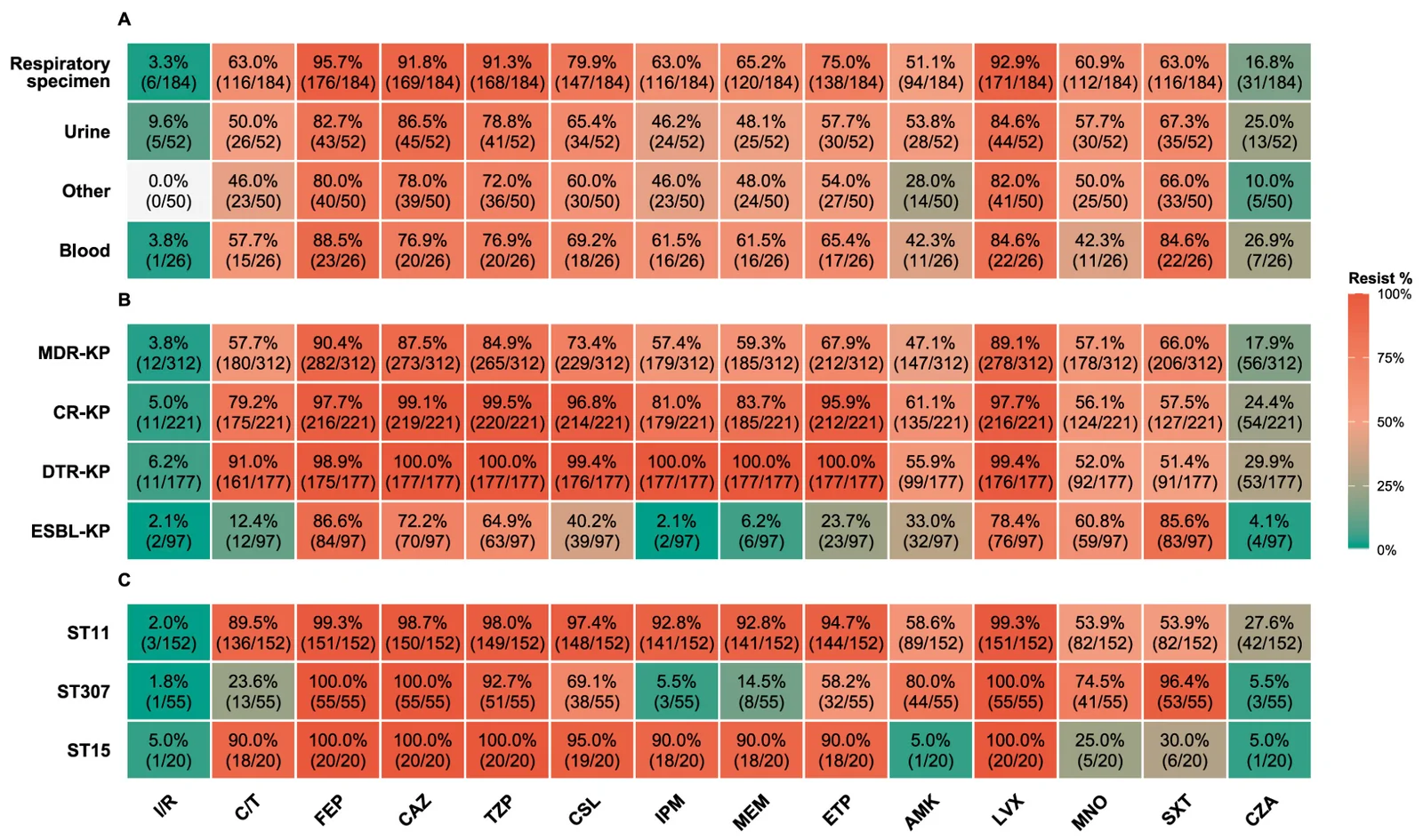
Comprehensive antimicrobial resistance profiles of multidrug-resistant *Klebsiella pneumoniae* (MDR-KP) isolates. Heatmaps illustrate the resistance rates to antimicrobial agents categorized by (**A**) specimen source, (**B**) resistance phenotype, and (**C**) sequence type (STs). Only STs with a sample size of ≥10 are included in panel (**C**). The color gradient represents the resistance rate, ranging from light gray (0%) to dark red (100%). Numerical labels within each tile denote the resistance percentage and the absolute ratio of resistant strains to the total number of tested isolates (*n*/total).

**Figure 4:**
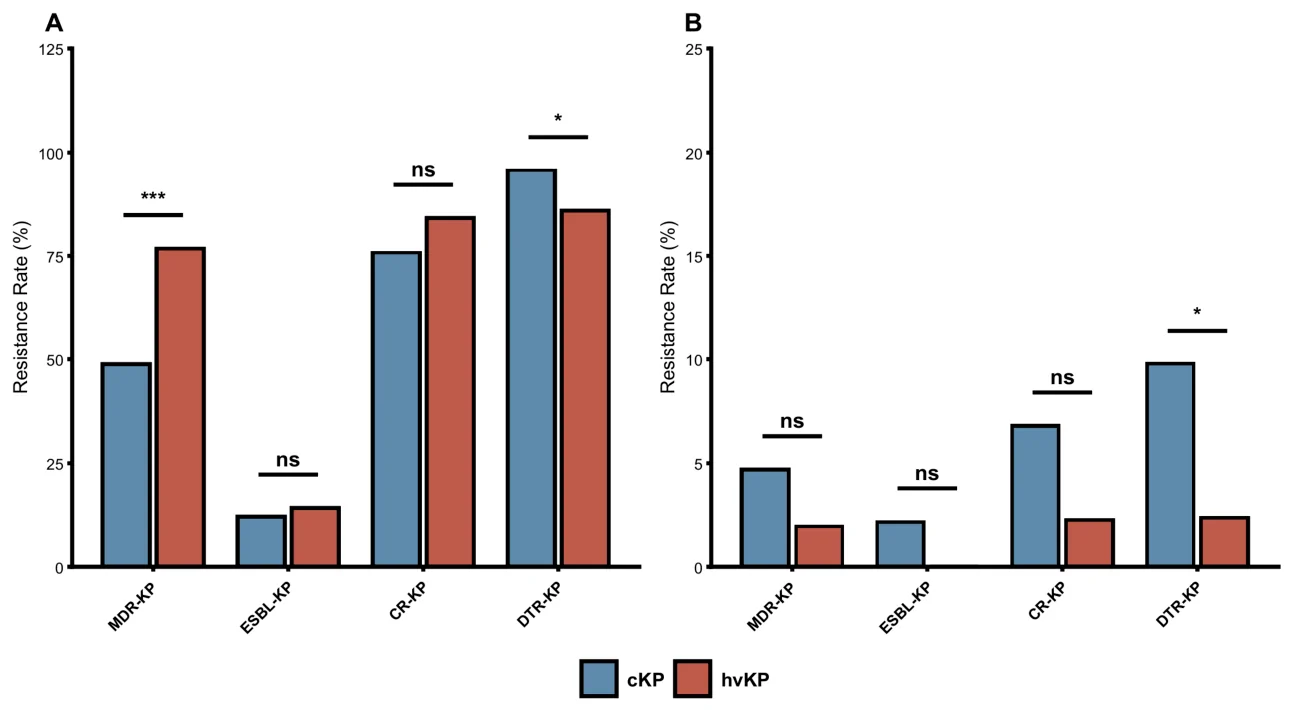
Comparative analysis of antimicrobial resistance rates between classic and hypervirulent *Klebsiella pneumoniae* (cKP and hvKP) across different phenotypic subgroups. (**A**) Resistance rates of C/T in cKP and hvKP. (**B**) Resistance rates of I/R in cKP and hvKP. Isolates are categorized into four subgroups: MDR-KP (Overall, *N*= 312), ESBL-KP (*n* = 97), CR-KP (*n* = 221), and DTR-KP (*n* = 177). Bar heights represent the percentage of resistant isolates. The steel blue bars represent classic *K. pneumoniae* (cKP), while the terra cotta bars represent hypervirulent *K. pneumoniae* (hvKP). *p*-values are indicated above the brackets: ***, *p* < 0.001; *, *p* < 0.05; ns, not significant (*p* ≥ 0.05).

**Table 1:** Antimicrobial susceptibility testing results for MDR-KP strains.

Antibiotic	No. Tested (*N*)	R, *n* (%)	I/SDD, *n* (%)	S, *n* (%)
I/R	312	12 (3.85%)	20 (6.41%)	280 (89.74%)
C/T	312	180 (57.69%)	30 (9.62%)	102 (32.69%)
FEP	312	282 (90.38%)	6 (1.92%)	24 (7.69%)
CAZ	312	273 (87.50%)	16 (5.13%)	23 (7.37%)
TZP	312	265 (84.94%)	16 (5.13%)	31 (9.94%)
CSL	312	229 (73.40%)	17 (5.45%)	66 (21.15%)
IPM	312	179 (57.37%)	1 (0.32%)	132 (42.31%)
MEM	312	185 (59.29%)	5 (1.60%)	122 (39.10%)
ETP	312	212 (67.95%)	8 (2.56%)	92 (29.49%)
AMK	312	147 (47.12%)	0 (0.00%)	165 (52.88%)
LVX	312	278 (89.10%)	28 (8.97%)	6 (1.92%)
MNO	312	178 (57.05%)	104 (33.33%)	30 (9.62%)
SXT	312	206 (66.03%)	0 (0.00%)	106 (33.97%)
CZA	312	56 (17.95%)	0 (0.00%)	256 (82.05%)

R, resistant; I, intermediate; SDD, susceptible-dose dependent; S, susceptible; I/R, imipenem/relebactam; C/T, ceftolozane/tazobactam; FEP, cefepime; CAZ, ceftazidime; TZP, piperacillin/tazobactam; CSL, cefoperazone/sulbactam; IPM, imipenem; MEM, meropenem; ETP, ertapenem; AMK, amikacin; LVX, levofloxacin; MNO, minocycline; SXT, trimethoprim-sulfamethoxazole; CZA, ceftazidime-avibactam.

**Table 2:** Univariable and multivariable logistic regression analysis of factors associated with C/T resistance in MDR-KP.

Variable	OR (95% CI)	*p* Value	aOR (95% CI)	*p* Value
hvKP	3.46 (2.05–6.03)	<0.001	0.37 (0.10–1.20)	0.117
ST11	47.08 (22.23–107.65)	<0.001	16.18 (3.56–77.11)	<0.001
ST15	49.85 (12.49–338.67)	<0.001	19.43 (2.62–189.83)	0.005
ST307	1.71 (0.72–4.08)	0.218	3.87 (1.23–14.03)	0.026
*bla*_KPC__-__2_	62.33 (30.90–137.10)	<0.001	25.98 (7.07–113.35)	<0.001
*bla*_NDM__-__1_	7.71 (1.45–142.32)	0.053	115.46 (15.70–2529.49)	<0.001
ESBL genes	1.57 (0.94–2.63)	0.084	0.99 (0.40–2.45)	0.990
Respiratory source	1.71 (1.08–2.70)	0.022	1.71 (0.73–4.10)	0.217

aOR, adjusted odds ratio; CI, confidence interval; The multivariable model included all variables listed, with cKP, other STs, non-respiratory specimens and gene-negative isolates as reference categories. Events per variable (EPV) was 22.5 in the multivariable model.

**Table 3:** Resistance rates of I/R and C/T across different β-lactamase-producing subgroups.

Subgroup	Total (*N*)	I/R Resistant *n* (%)	C/T Resistant *n* (%)
*bla*_KPC-2_-producing isolates	164	4 (2.4%)	153 (93.3%)
*bla*_CTX-M-65_ + bla_KPC-2_	50	2 (4.0%)	45 (90.0%)
*bla*_KPC-2_ alone	31	0 (0.0%)	28 (90.3%)
*bla*_CTX-M-65_ + *bla*_SHV-12_ + *bla*_KPC-2_	25	1 (4.0%)	24 (96.0%)
*bla*_CTX-M-65_ + *bla*_CMY-42_ + *bla*_KPC-2_	16	0 (0.0%)	16 (100.0%)
*bla*_CTX-M-15_ + *bla*_KPC-2_	15	0 (0.0%)	15 (100.0%)
*bla*_SHV-12_ + *bla*_KPC-2_	15	0 (0.0%)	14 (93.3%)
*bla*_KPC-2_ + *bla*_NDM-1_	3	1 (33.3%)	2 (66.7%)
Other *bla*_KPC-2_ combinations	9	0 (0.0%)	9 (100.0%)
*bla*_NDM-1_-producing isolates	11	6 (54.5%)	10 (90.9%)
ESBL-producing isolates	97	2 (2.1%)	12 (12.4%)
*bla*_CTX-M-15_	29	0 (0.0%)	3 (10.3%)
*bla*_CTX-M-55_	26	0 (0.0%)	5 (19.2%)
*bla*_CTX-M-3_	19	1 (5.3%)	1 (5.3%)
*bla*_CTX-M-14_	9	0 (0.0%)	0 (0.0%)
*bla*_CTX-M-27_	5	1 (20.0%)	1 (20.0%)
Other ESBLs	9	0 (0.0%)	2 (22.2%)
No ESBL/Carbapenemase Genes *	43	1 (2.3%)	7 (16.3%)

I/R, imipenem-relebactam; C/T, ceftolozane/tazobactam; ESBL, extended-spectrum β-lactamase; R, resistant. * indicates isolates with no detectable extended-spectrum β-lactamase or carbapenemase genes. Three *bla*_KPC-2_/*bla*_NDM-1_ co-carriers were included in both the *bla*_KPC-2_ and *bla*_NDM-1_-producing isolates.

## Data Availability

The whole-genome sequencing data generated and analyzed in this study have been deposited in the National Center for Biotechnology Information (NCBI) database. Genome sequences for isolates previously generated are available under BioProject accession number PRJNA1219236. The genome sequences for additional isolates generated in the present study are available under BioProject accession number PRJNA1484769.

## Supplementary Materials

The following supporting information can be downloaded at: https://www.mdpi.com/article/10.3390/antibiotics15100947/s1, Figure S1: Distribution of Resistance and Virulence Genes Categorized by Sequence Types; Figure S2: Comparison of antimicrobial resistance landscapes between cKP and hvKP subsets across clinical categories; Table S1: Statistical comparison of resistance rates for C/T and I/R between cKP and hvKP across various phenotypic subgroups; Table S2: Univariable and multivariable Firth logistic regression analysis of factors associated with I/R resistance in MDR-KP; Table S3: Characteristics of 12 I/R resistant *Klebsiella pneumoniae* isolates.

## Abbreviations

The following abbreviations are used in this manuscript:

AMK	Amikacin
aOR	Adjusted odds ratio
BL/BLI	β-lactam/β-lactamase inhibitor
C/T	Ceftolozane-tazobactam
CAZ	Ceftazidime
cKP	Classic Klebsiella pneumoniae
CLSI	Clinical and Laboratory Standards Institute
CR-hvKP	Carbapenem-resistant hypervirulent *Klebsiella pneumoniae*
CR-KP	Carbapenem-resistant *Klebsiella pneumoniae*
CRE	Carbapenem-resistant *Enterobacterales*
CSL	Cefoperazone-sulbactam
CZA	Ceftazidime-avibactam
DTR-KP	Difficult-to-treat resistant *Klebsiella pneumoniae*
ESBL	Extended-spectrum β-lactamase
ETP	Ertapenem
FEP	Cefepime
hvKP	Hypervirulent *Klebsiella pneumoniae*
I/R	Imipenem-relebactam
IPM	Imipenem
LIS	Laboratory information system
LVX	Levofloxacin
MDR-KP	Multidrug-resistant *Klebsiella pneumoniae*
MEM	Meropenem
MIC	Minimum inhibitory concentration
MNO	Minocycline
ST	Sequence type
SXT	Trimethoprim-sulfamethoxazole
TZP	Piperacillin-tazobactam
