# Supplementary material for: In Vitro Activity of Imipenem/Relebactam and Ceftolozane/Tazobactam Against Multidrug-Resistant Klebsiella pneumoniae: A Retrospective Single-Center Study in China
Source: Antibiotics (Basel). 2026 Sep 23;15(10):947. doi: 10.3390/antibiotics15100947 (PMC13700561; doi:10.3390/antibiotics15100947)
Supplement: Supplementary file 1 [file antibiotics-15-00947-s001.zip › antibiotics-4545368-supplementary.pdf]

Supplemental Material

Table S1. Statistical comparison of resistance rates for C/T and I/R between cKP and hvKP across various phenotypic subgroups.

Subgroup	Antibiotic	cKP (n/N, %)	hvKP (n/N, %)	*P* Value
MDR-KP	C/T	104/213, 48.8%	76/99, 76.8%	<0.001
MDR-KP	I/R	10/213, 4.7%	2/99, 2.0%	0.351
DTR-KP	C/T	88/92, 95.7%	73/85, 85.9%	0.024
DTR-KP	I/R	9/92, 9.8%	2/85, 2.4%	0.041
CR-KP	C/T	101/133, 75.9%	74/88, 84.1%	0.144
CR-KP	I/R	9/133, 6.8%	2/88, 2.3%	0.207
ESBL-KP	C/T	11/90, 12.2%	1/7, 14.3%	1.000
ESBL-KP	I/R	2/90, 2.2%	0/7, 0.0%	1.000

Table S2. Univariable and multivariable Firth logistic regression analysis of factors associated with I/R resistance in MDR-KP.

Variable	OR (95% CI)	P value	aOR (95% CI)	*P* value
hvKP	0.50 (0.09-1.76)	0.297	0.52 (0.07-3.23)	0.488
ST11	0.25 (0.06-0.85)	0.026	3.70 (0.38-31.53)	0.240
ST15	0.81 (0.08-4.02)	0.812	7.04 (0.51-69.47)	0.129
ST307	0.29 (0.03-1.37)	0.126	0.23 (0.01-1.95)	0.186
bla_{KPC-2}	0.46 (0.13-1.43)	0.184	0.24 (0.05-1.35)	0.099
bla_{NDM-1}	53.73 (13.85-226.72)	<0.001	56.62 (11.46-383.20)	<0.001
ESBL genes	0.63 (0.20-2.25)	0.454	0.55 (0.12-2.86)	0.454
Respiratory source	0.69 (0.22-2.14)	0.508	0.94 (0.25-3.53)	0.925

Firth's penalized logistic regression was used, and 95% CIs were estimated using profile penalized likelihood. EPV was 1.5; results should be interpreted as exploratory. The multivariable model included all variables listed, with cKP, other STs, non-respiratory specimens and gene-negative isolates as reference.

Table S3. Characteristics of 12 I/R resistant *Klebsiella pneumoniae* isolates.

ID	Specimen source	I/R (mg/L)	ST	Virulence genes	ESBL	Carbapenemase	OmpK mutations
PE14538	Blood	4	ST11	-	*bla*$_{CTX-M-65}$	*bla*$_{KPC-2}$	OmpK35-17%, OmpK36-9%
PE351375	Respiratory specimen	16	ST11	iucA, rmpA2	*bla*$_{CTX-M-65}$, *bla*$_{SHV-12}$	*bla*$_{KPC-2}$	OmpK35-17%, OmpK36GD
PE361437	Respiratory specimen	32	ST11	-	*bla*$_{CTX-M-65}$	*bla*$_{KPC-2}$	OmpK35-17%, OmpK36GD
PE341327	Respiratory specimen	12	ST147	-	*bla*$_{CTX-M-15}$	*bla*$_{NDM-1}$	OmpK35-7%, OmpK36GD
PE391594	Urine	12	ST147	-	*bla*$_{CTX-M-15}$	*bla*$_{NDM-1}$	OmpK35-7%, OmpK36GD
PEKP2022	Respiratory specimen	32	ST15	-	*bla*$_{CTX-M-3}$	-	-
21P1120	Respiratory specimen	16	ST20	-	-	-	-
PEKP3107	Urine	4	ST218	iucA, iroB, peg-344, rmpA2, rmpA	-	*bla*$_{NDM-1}$	-
PE7171	Urine	4	ST22	-	-	*bla*$_{KPC-2}$, *bla*$_{NDM-1}$	-
PE11	Urine	12	ST307	-	-	*bla*$_{NDM-1}$	OmpK35-21%, OmpK36GD
PEKP2013	Urine	16	ST37	-	*bla*$_{CTX-M-27}$	-	-
PE341351	Respiratory specimen	4	ST873	-	*bla*$_{CTX-M-14}$	*bla*$_{NDM-1}$	-

No additional *bla*$_{KPC-2}$ variants were identified among *bla*$_{KPC-2}$-producing isolates.

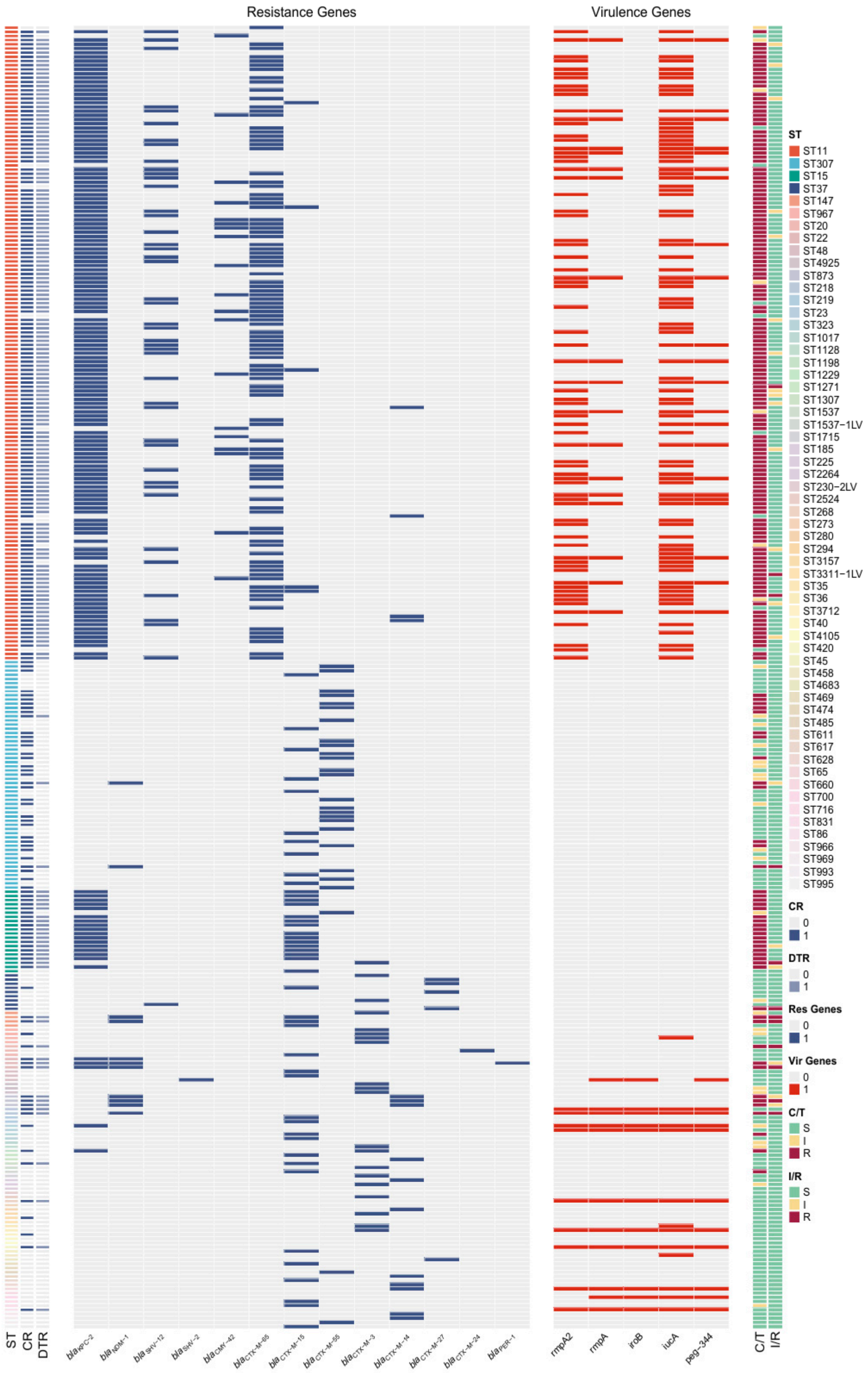

Figure S1. Distribution of Resistance and Virulence Genes Categorized by Sequence Types.

The heatmap displays the presence (colored) or absence (light gray) of key resistance genes (left) and virulence factors (right) across all isolates, ordered by sequence types (STs). Rows represent individual isolates, and columns represent specific genetic markers.

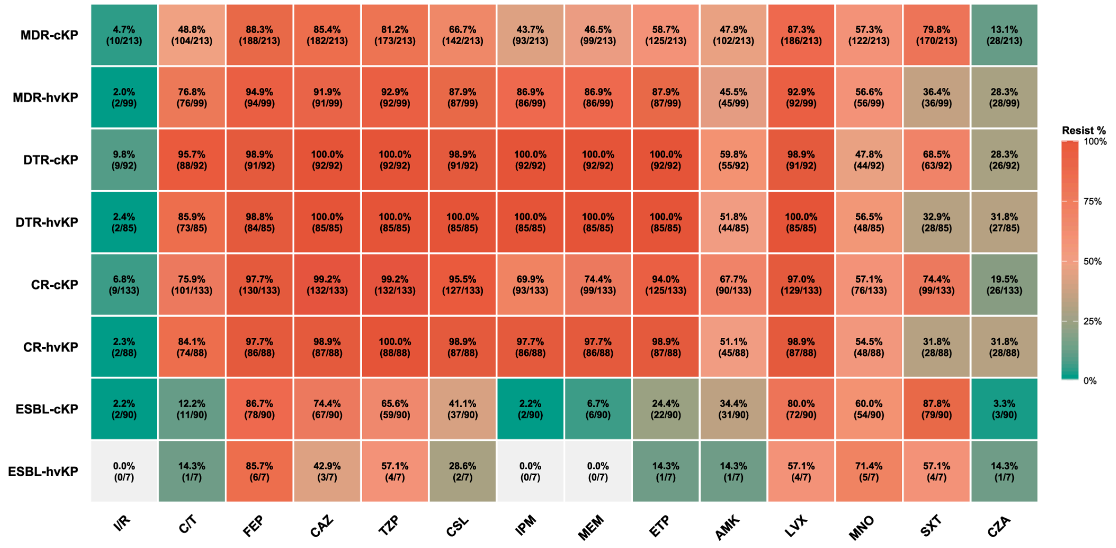

Figure S2. Comparison of antimicrobial resistance landscapes between cKP and hvKP subsets across clinical categories.

The heatmap illustrates the antimicrobial resistance profiles of 312 *K. pneumoniae* isolates, stratified by both resistance phenotypes and virulence status. Subsets include cKP and hvKP within the groups of MDR, DTR, CR, and ESBL isolates.
